# A Modular, Dynamic, DNA-Based Platform for Regulating Cargo Distribution and Transport between Lipid Domains

**DOI:** 10.1021/acs.nanolett.0c04867

**Published:** 2021-03-18

**Authors:** Roger Rubio-Sánchez, Simone Eizagirre Barker, Michal Walczak, Pietro Cicuta, Lorenzo Di Michele

**Affiliations:** † Biological and Soft Systems, Cavendish Laboratory, University of Cambridge, JJ Thomson Avenue, Cambridge CB3 0HE, United Kingdom; ‡ Molecular Sciences Research Hub, Department of Chemistry, Imperial College London, London W12 0BZ, United Kingdom

**Keywords:** DNA nanotechnology, lipid phase separation, lipid domains, partitioning, artificial cells, synthetic membranes, biomimicry

## Abstract

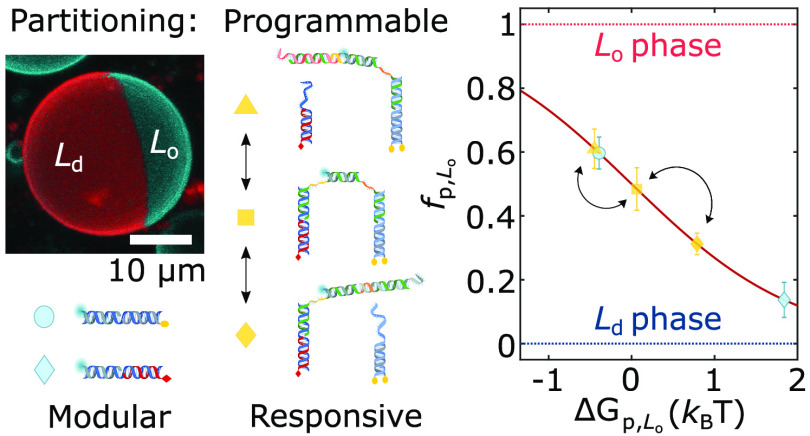

Cell membranes regulate the distribution of biological machinery between phase-separated lipid domains to facilitate key processes including signaling and transport, which are among the life-like functionalities that bottom-up synthetic biology aims to replicate in artificial-cellular systems. Here, we introduce a modular approach to program partitioning of amphiphilic DNA nanostructures in coexisting lipid domains. Exploiting the tendency of different hydrophobic “anchors” to enrich different phases, we modulate the lateral distribution of our devices by rationally combining hydrophobes and by changing nanostructure size and topology. We demonstrate the functionality of our strategy with a bioinspired DNA architecture, which dynamically undergoes ligand-induced reconfiguration to mediate cargo transport between domains via lateral redistribution. Our findings pave the way to next-generation biomimetic platforms for sensing, transduction, and communication in synthetic cellular systems.

Biological membranes are highly heterogeneous, containing up to 20% of the protein content of a cell and featuring hundreds of different lipid species.^1,2^ Such a degree of complexity evolved alongside the myriad of biological processes hosted and regulated by membranes, which include signaling, adhesion, trafficking, motility, and division.^3^ Many of these functionalities rely on lateral colocalization of membrane proteins^4^ required for the emergence of signaling hubs,^5,6^ focal adhesions,^7^ and assemblies regulating membrane architecture to promote endo- and exocytosis^8^ and cell division.^9^

Cells have evolved a variety of active and passive mechanisms to regulate the local composition of their membranes, overcoming the extreme molecular heterogeneity.^10−12^ Among these, proteolipid phase separation is thought to play an important role in signaling and signal transduction.^13^ In this process, nanoscale domains or *rafts* are believed to emerge, rich in sphingomyelins and sterols, which are able to recruit or exclude membrane proteins based on their different affinities for raft and non-raft environments.^14,15^

Bottom-up synthetic biology aims at replicating functionalities typically associated with biological cells in microrobots designed *de novo* or “artificial cells”.^16−18^ Just like their biological counterparts, many artificial-cell designs rely on semipermeable membranes for their compartmentalization requirements,^19−21^ which can be constructed from polymers^22^ and proteopolymer systems,^23,24^ colloids^25,26^ and, more often, synthetic lipid bilayers.^21^ However, with some remarkable exceptions,^27−31^ reviewed in ref (32), the membranes of artificial cells are often passive enclosures, lacking the complex functionalities of biological interfaces. A precise control over the local molecular makeup of synthetic lipid bilayers is therefore highly desirable and a necessary stepping stone for the development of ever more sophisticated life-like responses in artificial cells.

DNA nanotechnology has demonstrated great potential as a means of creating responsive nanostructures that emulate biological architectures and functionalities and are becoming increasingly popular constituents of artificial cellular systems.^33,34^ In many cases, biomimetic DNA nanostructures, rendered amphiphilic by hydrophobic tags, have been interfaced to synthetic lipid membranes to replicate the response of cell-membrane machinery. Examples include DNA architectures mediating artificial cell adhesion and tissue formation,^35−41^ regulating transport,^42,43^ enabling signal transduction,^44^ and tailoring membrane curvature.^45,46^

Importantly, amphiphilic DNA nanostructures have been demonstrated to undergo preferential partitioning when anchored to phase-separated synthetic bilayers, an effect that is reminiscent of membrane-protein partitioning in rafts.^47−49^ The preference of DNA nanostructures for different lipid phases and their degree of partitioning have been shown to depend on the chemical identity of the hydrophobic anchors, membrane lipid composition, temperature, and solvent conditions.^40,50^ For instance, in ternary model membranes of 1,2-dioleoyl-*sn*-glycero-3-phosphocholine (DOPC)/1,2-dipalmitoyl-*sn*-glycero-3-phosphocholine (DPPC)/cholesterol displaying coexistence of liquid ordered (L_o_) and liquid disordered (L_d_) phases,^1,51,52^ DNA constructs bearing a single cholesteryl-tri(ethylene glycol) (TEG) anchor show a weak preference for L_o_, while duplexes end-functionalized with two cholesteryl-TEG anchors partition in L_o_ more prominently.^53,54^ Conversely, oligonucleotides functionalized with α-tocopherol preferentially localize within the L_d_ phases of ternary 1-palmitoyl-2-oleoyl-*sn*-glycero-3-phosphocholine (POPC)/sphingomyelin (SM)/cholesterol membranes.^55,56^ Moreover, dynamic control over the partitioning has also been exemplified with DNA nanodevices that redistribute between lipid phases following enzymatic cleavage^57^ or changes in ionic strength.^58^

The ability to influence partitioning of membrane-anchored DNA constructs makes them promising tools for engineering the molecular makeup and functionality of artificial cell membranes. Yet, we have effectively only started to scratch the surface of the massive design space of amphiphilic DNA nanostructures that, alongside the chemical identity of the hydrophobes, can be freely engineered with respect to their size, topology, and stimuli responsiveness.

Here we introduce a modular platform that fully exploits the design versatility of DNA nanotechnology to construct nanostructures whose ability to partition in the domains of phase-separated synthetic membranes can be precisely programmed. Besides enabling static membrane patterning, the nanodevices can be dynamically reconfigured upon exposure to molecular cues, triggering redistribution between domains and unlocking synthetic pathways for cargo transport, signaling and morphological adaptation in synthetic cellular systems.

Our nanostructures were constructed from synthetic DNA oligonucleotides, some of which were covalently linked to hydrophobic moieties to mediate anchoring to the membrane. Fluorescent tags (Alexa488, unless stated otherwise) were also included to monitor the distribution of the nanostructures via confocal microscopy. As depicted in Figure 1 (left), we decorated the outer leaflet of phase-separated giant unilamellar vesicles (GUVs) with the DNA constructs. Unless specified otherwise, GUVs were prepared from ternary lipid mixtures (DOPC/DPPC/cholestanol) to display L_d_–L_o_ coexistence. To enable fluorescence imaging, the vesicles were doped at 0.8% molar ratio with TexasRed-DHPE, which preferentially labels the L_d_ phase. At room temperature, the GUVs readily de-mixed into two macroscopic L_o_ and L_d_ domains. The resulting Janus-like morphology, shown in Figure 1 (right), enabled the facile visualization and quantitation of the lateral distribution of the nanostructures.

**Figure 1:**
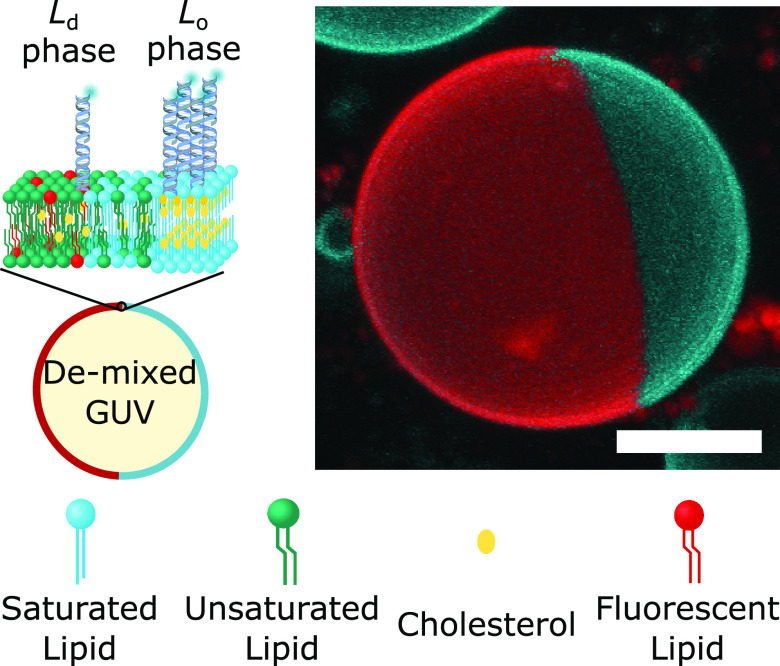
Membrane-anchored DNA nanostructures display preferential partitioning between the phases of de-mixed giant unilamellar vesicles. (left) Schematic representation of a multicomponent giant unilamellar vesicle (GUV), prepared with saturated and unsaturated lipids mixed with sterols, displaying liquid-ordered (L_o_) and liquid-disordered (L_d_) phase coexistence. The outer leaflet of the membrane is functionalized with amphiphilic DNA nanostructures, exemplified here with constructs bearing two cholesterol anchors, which enrich their preferred (L_o_) phase. (right) 3D view of a de-mixed GUV from confocal *z*-stack as obtained from Volume viewer (FIJI^59^) using maximum projection. DNA nanostructures (cyan) partition to the L_o_ domain, while the L_d_ phase (red) is labeled with TexasRed-DHPE. Scale bar = 10 μm.

To this end, equatorial confocal micrographs of the GUVs were collected and analyzed with a custom-built image processing pipeline, as described in detail in the SI (see Methods, and the associated Figures S1 and S2), which determined the average fluorescence intensities of the constructs in the two phases: *I*_L_o__ and *I*_L_d__. From these values, fractional intensities *f*_p,L_o_(L_d_)_ = *I*_L_o_(L_d_)_/(*I*_L_o__ + *I*_L_d__) were extracted. Throughout this letter, we refer to the fractional fluorescence intensity of nanostructures in the L_o_ phase, *f*_p,L_o__, to gauge their partitioning tendency. Förster resonance energy transfer (FRET) between Alexa488 on the DNA and TexasRed on the lipids, alongside fluorescent-signal cross-talk, could in principle bias *f*_p,L_o__ in certain partitioning states. To rule out this possibility, we performed dedicated experiments to determine the impact of both potential artifacts, as well as controls on GUVs that lack the TexasRed fluorophore, thus altogether eliminating the possible source of bias. Data in Figure S3, and the associated Supplementary Discussion 1, confirm that FRET and fluorescence cross-talk carry a negligible impact on *f*_p,L_o__.

Assuming that the recorded fluorescence intensities are proportional to nanostructure concentrations, a partitioning free energy can be calculated as Δ*G*_p,L_o__ = −*k*_B_*T* log(*f*_p,L_o__/*f*_p,L_d__). The latter is defined as the free energy change associated with relocating a single construct from the L_d_ to the L_o_ phase.

First, we applied our data-analysis pipeline on simple DNA duplexes featuring well characterized single cholesterol-TEG (sC) or single tocopherol (sT) anchors, as summarized in Figure 2. Expectedly, while sC anchors led to a marginal L_o_-preference (*f*_p,L_o__^sC^ ≈ 0.6, Δ*G*_p,L_o__^sC^ ≈ −0.4*k*_B_*T*), nanostructures featuring sT strongly partitioned in L_d_ (*f*_p,L_o__^sT^ ≈ 0.14, Δ*G*_p,L_o__^sT^ ≈ 1.9*k*_B_*T*).

**Figure 2:**
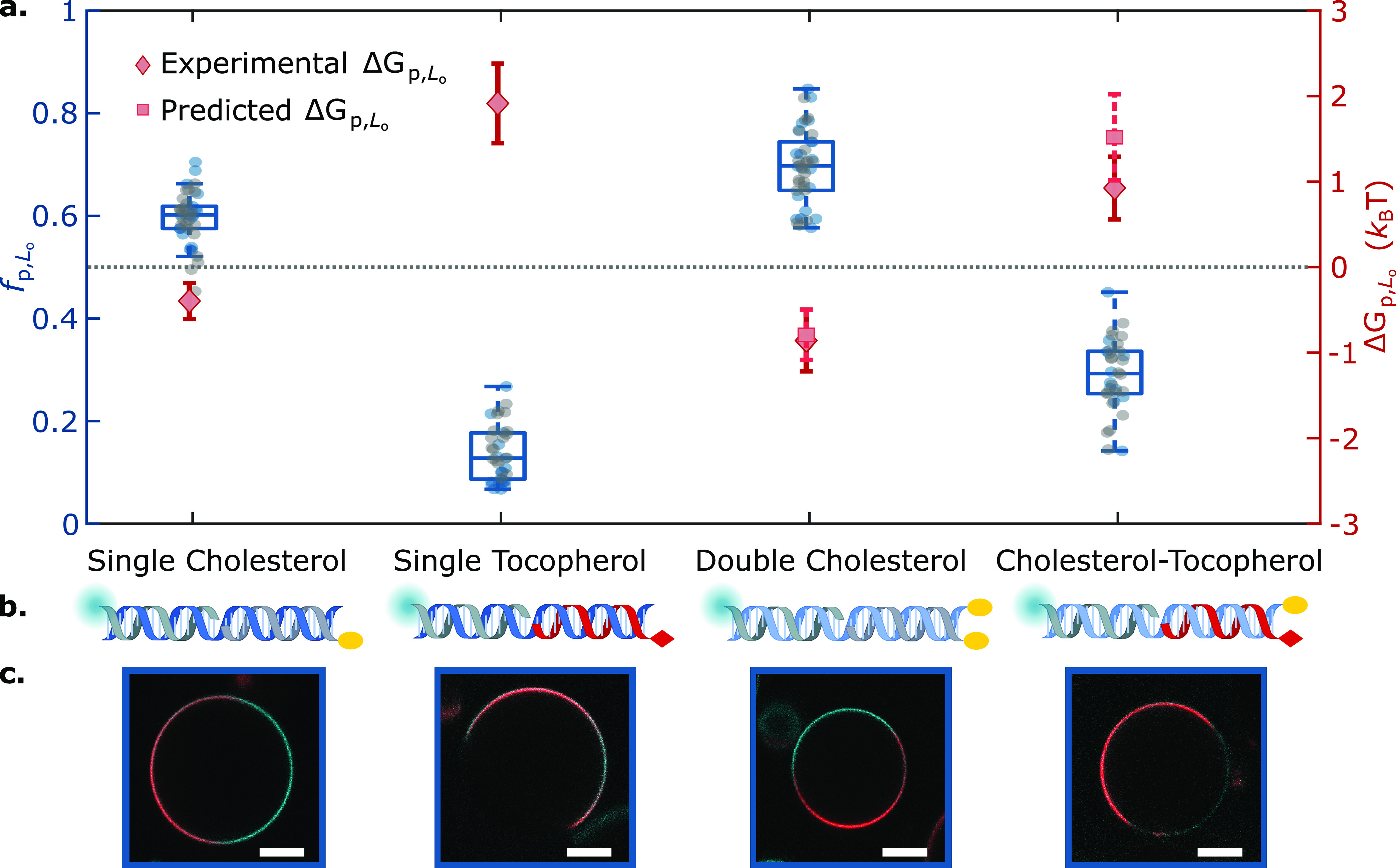
Lateral distribution of DNA nanostructures depends on anchor chemistry and combination. (a) Fractional intensity (*f*_p,L_o__) and free energy of partitioning (Δ*G*_p,L_o__) of DNA nanostructures in L_o_, conveyed by box-scatter plots and lozenges respectively, for two individual repeats (in blue/gray) of DNA-decorated GUV populations featuring different anchoring motifs: single cholesterol-TEG (sC), double cholesterol-TEG (dC), single tocopherol (sT), or a combination of single cholesterol-TEG and single tocopherol (sC+sT). For dC and sC+sT, squares indicate the predicted free energy values for combined anchors, determined from eq 1 using measured values of Δ*G*_p,L_o__^sT^ and Δ*G*_p,L_o__^sC^. The dotted line indicates no partitioning (*f*_p,L_o__ = 0.5, Δ*G*_p,L_o__ = 0). (b) Schematic depictions of DNA duplexes bearing sC, sT, dC, or sC+sT. (c) Representative confocal micrographs of the DNA-functionalized GUVs. The L_d_ phase was labeled with TexasRed (red), and DNA constructs (cyan) were labeled with Alexa488. Scale bars = 10 μm.

Importantly, as highlighted in Figure S4, the size heterogeneity of electroformed GUVs does not affect the lateral distribution of DNA anchored species, as *f*_p,L_o__ does not correlate with vesicle radius.

The substantial difference in the partitioning behaviors induced by sT and sC traces a route to program lateral distribution by combining multiple or different hydrophobic moieties within the same nanostructure. With this modular “mix and match” approach, we can indeed think of reinforcing partitioning in either lipid phase by increasing the number of cholesterol or tocopherol moieties on our nanodevices, or to access intermediate partitioning states with nanostructures featuring both moieties.

In the absence of (anti) co-operative effects and at sufficiently low construct concentrations, the partitioning free energy of a nanostructure featuring multiple hydrophobic moieties should be additive in the contributions from individual anchors:
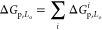
1where the index *i* runs over all the anchors in the construct.

The simple relationship in eq 1 can guide the design of multianchor motifs, and in Figure 2, we tested it on a duplex featuring two cholesterol-TEG anchors (dC). As expected, the dC motif displayed an enhanced preference for L_o_ domains compared to sC,^40,53^ with *f*_p,L_o__^dC^ ≈ 0.7. The measured partitioning free energy was Δ*G*_p,L_o__^dC^ ≈ −0.8*k*_B_*T*, nearly identical to twice that of sC nanostructures. This quantitative agreement with the prediction of eq 1 suggests that, at least in this specific case, anchor co-operativity and other nonadditive contributions negligibly affect partitioning.

When testing “chimeric” duplexes, bearing a tocopherol and a cholesterol anchor (sC+sT), we observed a pronounced preference for L_d_, consistent with the expectation that tocopherol should dominate in view of the stronger free energy shift associated with its partitioning (Figure 2, *f*_p,L_o__^sC+sT^ ≈ 0.3). Here, the free energy prediction from eq 1 slightly overestimated the measured value of Δ*G*_p,L_o__^sC+sT^ ≈ 0.9*k*_B_*T* (statistically significant difference, *p* = 8.5 × 10^–11^, using the nonparametric Mann–Whitney Wilconson Test). Since this nonadditive behavior was not observed for the dC construct, we argue that it may result from the distinct chemical nature of the anchors in sC+sT and the consequent differences in their interactions with the surrounding lipids.

To further challenge our modular design approach, we studied the partitioning behavior of the constructs in Figure 2 in a quaternary lipid mixture (DOPC/DPPC/cholestanol/cardiolipin). While this more complex mixture still displayed L_o_–L_d_ phase coexistence, the incorporation of the highly unsaturated cardiolipin has been shown to enhance L_o_-partitioning, owing to the increased lateral stress in the L_d_ phase.^40^ The data, summarized in Figure S5, largely confirmed the predictive power of eq 1, but some nonadditive deviations are highlighted. Specifically, we observed that applying the rule in eq 1 led to an overestimation of the nanostructures’ tendency to localize within the L_o_ for both dC and sC+sT motifs. The difference in magnitude between nonadditive free energy terms observed for ternary and quaternary lipid compositions is only partially surprising, as one would expect (anti) co-operative effects to be highly sensitive to the lipid microenvironment of the anchors. For instance, one may speculate that the cardiolipin-rich L_d_ phase in the quaternary mixture may be better able to accommodate larger inclusions like those generated by two-anchor motifs (dC, sC+sT), which may in turn help to relax the built-in lateral stress.^40^ This phenomenon may lead to a less pronounced L_o_ preference for two-anchor compared to single-anchor constructs.

Thanks to the design freedom of DNA nanotechnology, our modular strategy is not restricted to simple motifs with one or two anchors. We can indeed regard the duplex constructs in Figure 2 as “anchoring modules” and further combine them in larger nanostructures to expand the range of accessible partitioning states. For instance, as schematically depicted in Figure 3, two anchoring modules were coupled by simply connecting them with a transversal, fluorescently labeled linker duplex. For added conformational flexibility, 3-nt single-stranded (ss)DNA domains (α in Figure 3) were included between the hydrophobically modified and linker duplexes.

**Figure 3:**
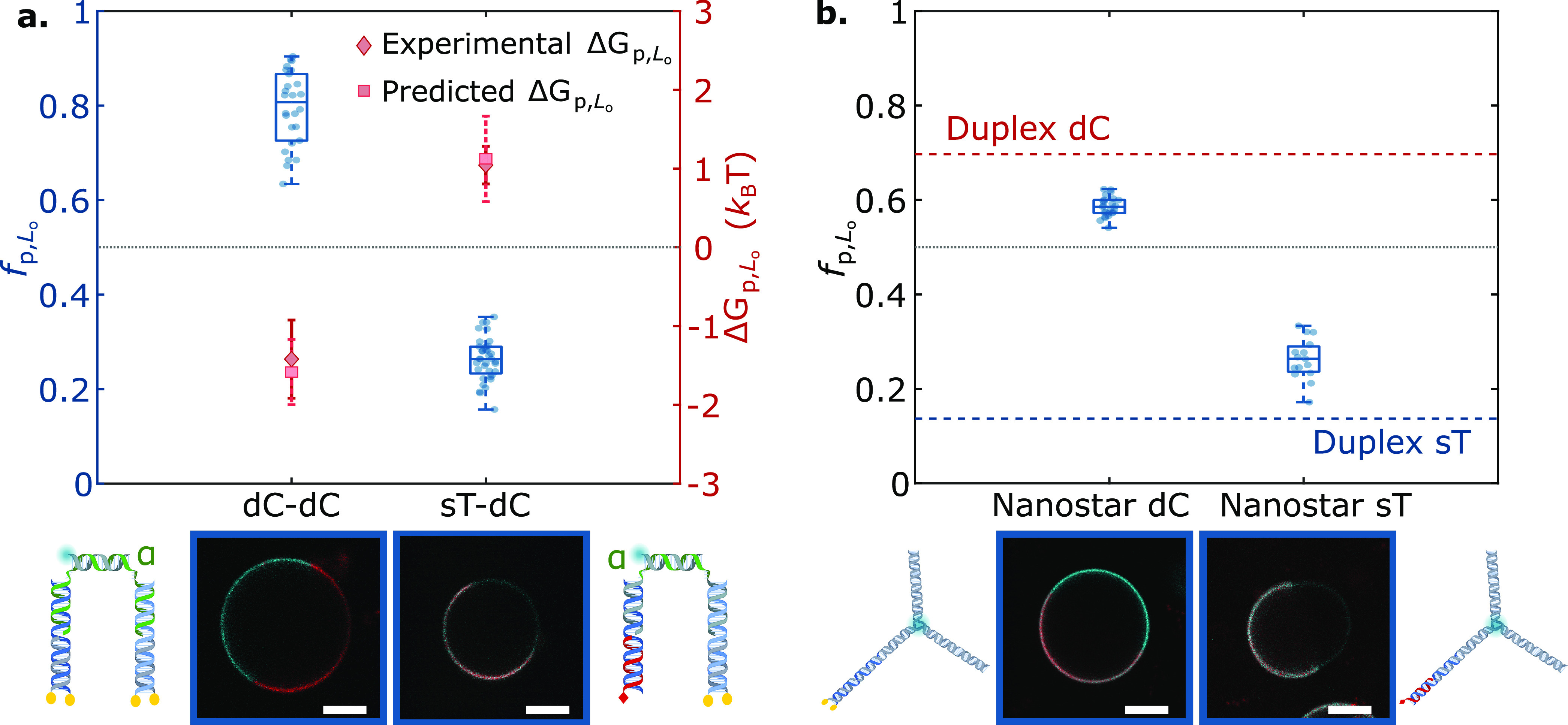
Anchor coupling and construct topology modulate DNA nanostructure partitioning. (a, top) Tunable lateral segregation of DNA architectures attained by coupling two sets of anchors: dC+dC and sT+dC. The partitioning behavior is demonstrated by the fractional intensity (*f*_p,L_o__, box-scatter plots) and free energy of partitioning (Δ*G*_p,L_o__, lozenges) in L_o_. Squares indicate the predicted free energy values for combined anchors, determined from eq 1 using measured values of Δ*G*_p,L_o__^sT^ and Δ*G*_p,L_o__^sC^ (Figure 2). (a, bottom) Schematic representations of the nanostructures and representative confocal micrographs of decorated GUVs. (b, top) Effect of nanostructure size on partitioning, shown via *f*_p,L_o__ box-scatter plots of DNA nanostars bearing single tocopherol (sT) or double-cholesterol (dC) anchors, compared against the mean *f*_p,L_o__ achieved by their duplex counterparts (dashed lines, red for dC, blue for sT). (b, bottom) Depiction of DNA nanostars alongside representative confocal micrographs of DNA-decorated GUVs. In plots in both panels a and b, the dotted line indicates no partitioning (*f*_p,L_o__ = 0.5, Δ*G*_p,L_o__ = 0). The L_d_ phase was labeled with TexasRed (red), and constructs (cyan) were labeled with Alexa488 (DNA–DNA complexes) or fluorescein (nanostars). Scale bars = 10 μm.

Two modular combinations were investigated: dC+dC and sT+dC. Notably, for both designs, the measured partitioning free energies could be quantitatively predicted by adding up the contributions of the individual anchor modules. The dC+dC construct, as expected, displays a very strong L_o_ preference, with Δ*G*_p,L_o__^dC+dC^ ≈ −1.4*k*_B_*T* and *f*_p,L_o__^dC+dC^ ≈ 0.8. Similarly to the case of the sC+sT constructs, the partitioning of sT+dC nanostructures was dominated by the strong L_d_ preference of the tocopherol, with Δ*G*_p,L_o__^sT+dC^ ≈ 1*k*_B_*T* and *f*_p,L_o__^sT+dC^ ≈ 0.26.

Despite the remarkable accuracy of eq 1 in predicting partitioning states in multianchor constructs, we highlighted nonadditive deviations. While some of these appear to correlate with the details of anchor and lipid chemistry (sC-sT in Figure 2 and Figure S5) and are thus difficult to control, other nonadditive contributions can potentially be exploited to fine-tune partitioning states around the baseline defined by anchor combination. For instance, the use of larger and more complex nanostructures may influence lateral segregation owing to steric or electrostatic interactions between the motifs. One may indeed expect that, for larger nanostructures, excluded volume effects may hinder accumulation in one specific phase, thus suppressing partitioning. Figure 3b summarizes the partitioning behavior of three-pointed DNA nanostars anchored to the bilayers using dC and sT modules. These motifs had roughly 4× the molecular weight of the smaller duplex architectures in Figure 2 and, indeed, systematically displayed a reduced partitioning tendency. Moreover, Figure S6 proves that the effect is not unique to the branched nanostructures, as linear duplexes anchored via dC also showed a general weakening in partitioning with increasing length. In further support to the hypothesis that steric nanostructure–nanostructure interactions may have an effect on partitioning, we performed measurements for smaller dC and sT duplexes over a wide range of (nominal) DNA-to-lipid molar ratios and thus of surface densities of the motifs, as summarized in Figure S7. We observed a near stationary *f*_p,L_o__ over a broad range of DNA/lipid ratios around the value used for all other experiments throughout this work (∼4 × 10^–4^). For both dC and sT constructs, however, *f*_p,L_o__ strongly deviated at high DNA/lipid ratios, approaching ∼0.5. Such a deviation hints at a saturation of the L_o_ and L_d_ phases and the consequent impossibility for the nanostructures to further accumulate in those domains. Saturation occurred at higher DNA/lipid ratios for sT compared to dC, and we argue that this difference may arise from differences in the overall membrane affinity of the anchors. Indeed, while dC membrane insertion is effectively irreversible,^60^ anchoring via sT may be weaker so that membrane-anchored sT duplexes coexist with a larger concentration of solubilized constructs, effectively reducing the surface density at fixed DNA/lipid ratios.

Our ability to program the domain partitioning of DNA constructs by linking different anchoring modules, as demonstrated in Figure 3, can be combined with the dynamic reconfigurability of DNA nanostructures to reversibly trigger redistribution of a fluorescent cargo within the GUVs’ surfaces upon exposure to molecular cues. We demonstrate this effect with a nanostructure featuring both dC and sT anchoring modules, similar to that shown in Figure 3 but in which the fluorescent dsDNA linker module (cargo) connecting the anchor duplexes can reversibly bind to or detach from either via toehold-mediated strand displacement,^61^ a mechanism that is reminiscent of two-component biological receptors undergoing ligand-induced dimerization.^5,6^ As sketched in Figure 4a, we initiated our system from a configuration in which the fluorescent cargo was attached to the sT anchor and thus localized in the L_d_ phase (State 1). Here, the fluorescent linker module was prevented from connecting to the dC anchoring module as its domain γ_1_^*^, complementary to γ_1_ on the dC module, was protected by an Antifuel_1_ strand of domain sequence δ_1_γ_1_α_1_^*^. Note that the *f*_p,L_o__ value recorded in this configuration matched exactly the one measured if the dC module was absent from the bilayer, marked by a dashed line in Figure 4b, thus confirming the absence of binding to the dC module. Antifuel_1_ could be displaced upon addition of Fuel_1_, leading to the exposure of γ_1_^*^ on the linker module and its binding to the dC module. Upon acquiring this configuration, State 2, the nanostructures shifted the cargo toward L_o_. Addition of Antifuel_2_, with sequence α_2_^*^γ_2_δ_2_, triggered the displacement of the linker from the sT module following a toeholding reaction initiated at domain α_2_, leading to the emergence of State 3 and a further cargo redistribution toward the L_o_ phase. Also in this configuration, the *f*_p,L_o__ value aligned to that measured in the absence of sT anchors, indicating a near-complete progression of the toeholding reaction (dot-dash line in Figure 4b). Finally, sequential addition of Fuel_2_ and Antifuel_1_ could reverse the systems’ configuration to States 2 and then 1. The last two steps pushed the fluorescent cargo back toward its initial L_d_ preference, but the *f*_p,L_o__ values remained slightly higher than those recorded at first. This incomplete reversibility may be due to partial inefficiencies in the reverse toeholding reactions^62^ or to a small thermodynamic unbalance that favors State 3 over State 1. Note that the system was allowed to fully equilibrate after the addition of fuel/antifuel strands prior to collecting the data in Figure 4, as demonstrated by the measurements acquired at intermediate time points and shown in Figure S9a. In turn, Figure S9b shows the time evolution of *f*_p,L_o__ for an individual GUV, in which the nanostructures transition from State 1 to State 2 upon fuel addition. The equilibration time scales of ∼5 min are likely limited by the diffusion of the fuel strand through the sample, given that, in order to prevent GUV drift and disruption, the fuel solution is gently added from the sample surface without any active mixing. The rates of toehold-mediated strand displacement and diffusion of the membrane-anchored constructs are expected to be comparatively faster (see discussion in the caption of Figure S9). The redistribution recorded for our nanodevices is comparable to those of biological membrane-anchored agents involved, for example, in the recruitment of receptors upon T-cell activation^63^ or clathrin-mediated endocytosis,^8,64^ both of which range between tens and hundreds of seconds. Finally, note that States 1 and 3 displayed a lower tendency to partition in L_d_ and L_o_ (respectively) compared to their sT and dC duplex analogues (Figure 2). The shift is likely a consequence of the greater steric encumbrance of the responsive motifs, discussed above with respect to Figure 3 and Figure S6.

**Figure 4:**
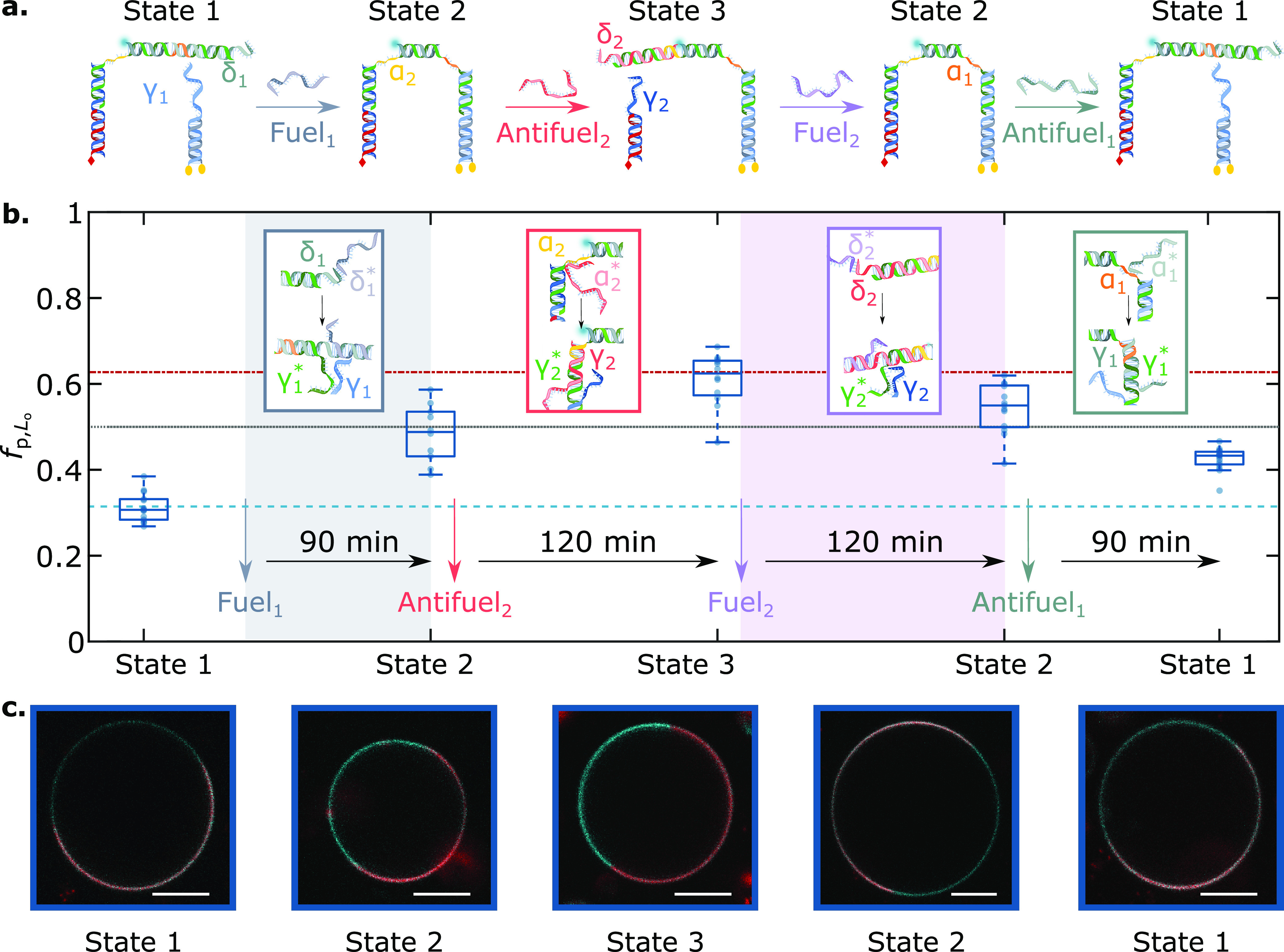
Toehold-mediated strand displacement enables cargo transport between lipid domains via lateral redistribution to programmed partitioned states: (a) Schematic depiction of the mechanism underpinning DNA nanostructure dimerization and cargo redistribution induced by the addition of Fuel/Antifuel strands, which exploit domains δ_1_, α_1_, δ_2_, and α_2_ as toeholds. The correct response of the molecular circuit was tested with agarose gel electrophoresis, as summarized in Figure S8. (b) Evolution of the partitioning tendency of the fluorescent DNA element (panel a), conveyed by *f*_p,L_o__ box-scatter plots, after a delay time for equilibration upon the addition of Fuel/Antifuel strands. The light blue dashed line marks the mean *f*_p,L_o__ of the cargo hybridized to the sT-bearing module and in the absence of the dC-anchoring module, while the red dash-dot line marks that of the cargo hybridized to the dC-bearing module and in the absence of the sT anchoring module. The dotted line indicates no partitioning (*f*_p,L_o__ = 0.5, Δ*G*_p,L_o__ = 0). (c) Representative confocal micrographs of DNA-functionalized GUVs over time, showcasing the distinct partitioned states attained by the system. The L_d_ phase was labeled with TexasRed (red), and DNA constructs were labeled with Alexa488 (cyan). Scale bars = 10 μm.

In summary, we introduced a modular approach to engineer the lateral distribution of amphiphilic DNA nanostructures between coexisting phases of synthetic membranes. We exploited the ability of individual cholesterol and tocopherol anchors to induce partitioning in L_o_ and L_d_, respectively, and combined them to produce an array of multianchor nanodevices that span a broad range of partitioning behaviors from ∼80% preference for L_o_ to ∼85% partitioning in L_d_, as summarized in Figure 5a. The comparison between measured partitioning free energies and those extracted from eq 1, shown in Figure 5b, proves that to a good approximation Δ*G*_p,L_o__ is additive in the contributions from individual anchors, thus offering a predictive design criterion. Small, nonadditive effects contribute to a different extent depending on anchor combinations and lipid-membrane composition, hinting at (anti) co-operative effects dependent on system-specific chemistry, while excluded-volume effects emerged for bulkier motifs and higher nanostructure concentrations.

**Figure 5:**
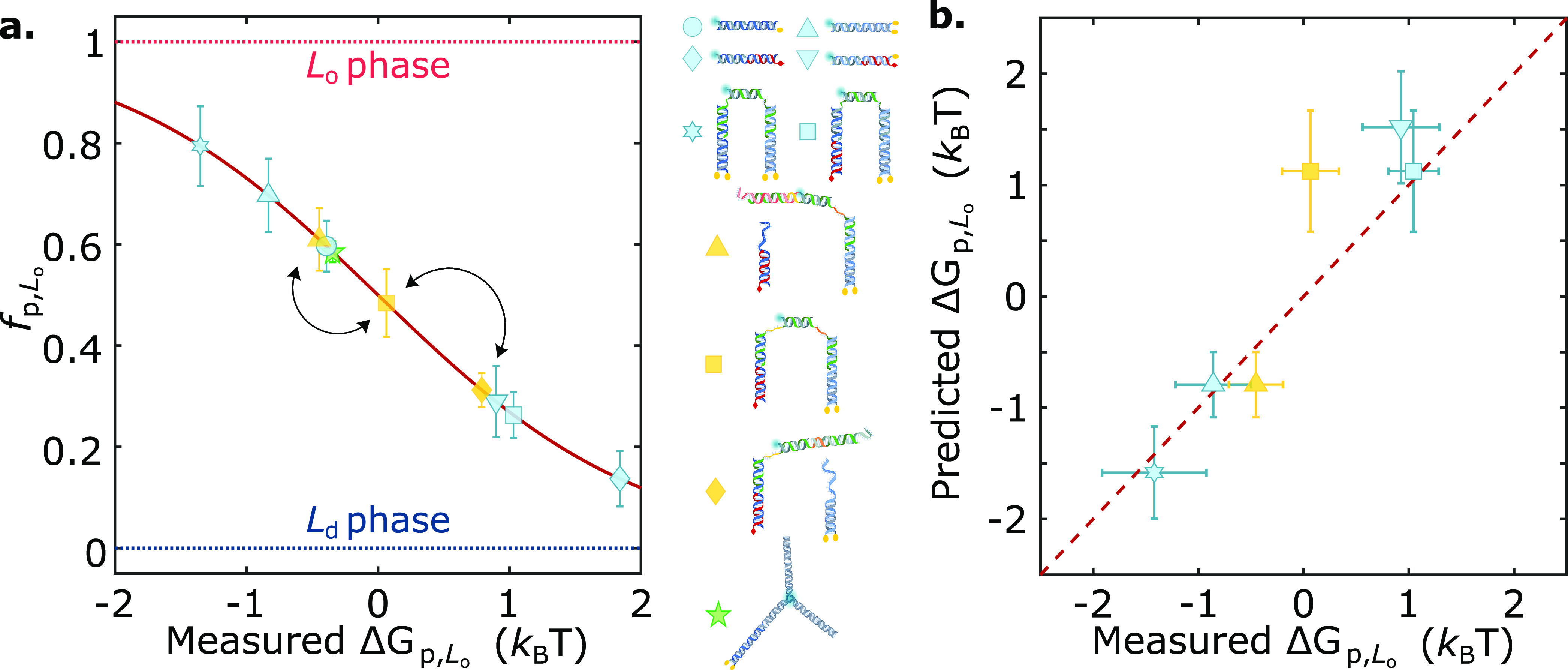
Static and dynamic engineering of partitioning states across the free-energy landscape by prescribing anchor combination and nanostructure morphology. (a) Summary graph demonstrating the mean *f*_p,L_o__ ± standard deviations of several membrane-associated DNA nanostructures as a function of their measured free energy of partitioning (Δ*G*_p,L_o__). The effect of anchor coupling, size, and geometry is showcased. Arrows connect programmed partitioned states achieved by the responsive nanodevice discussed in Figure 4. (b) Partitioning free energy of DNA nanostructures is to a good approximation additive in contributions from individual anchoring motifs, as shown for several multianchor nanostructures with the mean ± standard deviations of measured Δ*G*_p,L_o__ vs those predicted with eq 1.

The modularity of our design strategy enables dynamic control over the partitioning state by altering the anchor makeup of the nanostructures, as we showed with a proof-of-concept experiment in which fluorescent cargoes were reversibly transported across vesicle surfaces upon exposure to molecular cues. This strategy evokes that used by cells to control spatiotemporal localization of membrane proteins^47−49,65^ and can be further extended to respond to other physico-chemical triggers by including stimuli-sensitive motifs such as aptamers,^66^ DNAzymes,^67^ G-quadruplexes,^68^ pH-responsive motifs,^69^ and other functional DNA architectures.^70,71^

Our approach could be easily extended to include moieties other than cholesterol and tocopherol, such as alkyl chains,^40^ porphyrin,^72^ and azobenzene,^73^ which besides unlocking a broader range of partitioning states may also enable responsiveness to a wider spectrum of stimuli. Similar design principles could even be applied to membrane-associated entities different from (amphiphilic) DNA nanostructures, such as peripheral and integral proteins, to program their colocalization in membrane domains.^74,75^

Our platform paves the way for the development of next-generation biomimetic DNA devices for the bottom-up engineering of life-like behaviors in synthetic cellular systems, which could in the long term find application in high-tech therapeutics and diagnostic solutions. For instance, the stimuli-triggered reshuffling of membrane-bound objects between lipid phases can enable highly sought behaviors such as signal transduction, communication, and local membrane sculpting.^45^ Examples include stimuli-induced colocalization of *synthetic receptors* initiating artificial signaling cascades^44^ and herding of objects capable of influencing local membrane curvature, thus directing morphological restructuring such as tubular growth^76^ and exosome/endosome budding.^46,77,78^

## Supporting Information Available

Experimental methods, discussion of impact of fluorescence cross-talk and FRET, image analysis pipeline for measuring DNA-construct fluorescence intensity in equatorial confocal micrographs, circle fitting routine to correct for imperfect segmentation of low intensity equatorial membrane domains, impact of fluorescent lipid marker and GUV polydisperity on partitioning tendency, lateral distribution of duplex DNA nanostructures in quaternary (DOPC/DPPC/cardiolipin/chol) GUVs, lateral distribution of DNA nanostructures of increasing molecular weight, suppression of partitioning by high DNA/lipid ratio due to membrane saturation, DNA nanostructure reconfigurability via toeholding reactions, DNA nanostructure reconfiguration and redistribution, and DNA sequences of the nanostructures used throughout this work (PDF)
